# Risk Factors for Anterior Skull Base Injury in Endoscopic Sinus Surgery

**DOI:** 10.7759/cureus.49273

**Published:** 2023-11-23

**Authors:** Seiichiro Makihara, Kensuke Uraguchi, Tomoyuki Naito, Aiko Shimizu, Aya Murai, Takaya Higaki, Yohei Noda, Shin Kariya, Mitsuhiro Okano, Mizuo Ando

**Affiliations:** 1 Otolaryngology-Head and Neck Surgery, Okayama University Hospital, Okayama, JPN; 2 Otolaryngology-Head and Neck Surgery, Kagawa Rosai Hospital, Marugame, JPN; 3 Otolaryngology-Head and Neck Surgery, Fukuyama City Hospital, Fukuyama, JPN; 4 Otolaryngology, Kawasaki Medical School, Kurashiki, JPN; 5 Otolaryngology, School of Medicine, International University of Health and Welfare, Narita, JPN; 6 Otolaryngology-Head and Neck Surgery, Faculty of Medicine, Dentistry, and Pharmaceutical Sciences, Okayama University, Okayama, JPN

**Keywords:** posterior ethmoid roof, cerebrospinal fluid leak, anterior ethmoidal artery, gera classification, keros classification

## Abstract

Objectives

This retrospective study aimed to investigate the relationships between the Keros classification, the Gera classification, the vertical height of the posterior ethmoid roof (ER), and anterior ethmoidal artery (AEA) types in Japanese patients.

Methods

We investigated the computed tomography (CT) slices of paranasal sinuses (120 sides) of 60 patients; measured the cribriform plate (CP) depth, lateral lamella CP angle (LLCPA), and vertical height of the lateral ER from the hard palate (LERHP) at the coronal plane of the posterior ethmoidal artery (PEA); and reviewed the AEA types, whether floating or non-floating.

Results

CP depth was positively correlated with LLCPA (r=0.63; p<0.01) and the height of LERHP (r=0.19;* *p<0.05). The height of the LERHP in females was significantly lower than that in males. With increased CP depth, floating AEAs became prevalent (p<0.001).

Conclusion

In females, low height of the posterior ethmoid sinus roof, where cerebrospinal fluid (CSF) leaks occurred while penetrating the basal lamella, often existed; the heights positively correlated with the Keros classification in Japanese patients. The Keros and Gera classifications, AEA type, and posterior ER height do not individually constitute a complete risk assessment but may correlate, preventing major complications, such as CSF leak and orbital hemorrhage.

## Introduction

The conventional surgical treatment for chronic rhinosinusitis is endoscopic sinus surgery (ESS). However, in cases of functional ESS, minor complications, such as bleeding, infection, and synechia formation, arise in 1.1%-20.8% of cases [1,2], and significant complications, including cerebrospinal fluid (CSF) leak, intracranial injury involving major blood vessels or brain injury, and ocular injury, occur in 0%-1.5% of cases. Most major complications are associated with the ethmoid bone [1-3]. Incidental CSF leaks usually occur with negligent skull base puncturing during ESS. Their occurrence may decrease with the assistance of a navigation system but cannot be completely ruled out owing to the inaccuracy of the navigation system. A review of preoperative computed tomography (CT) slices is crucial in surgical planning and can detect anatomical variants that might lead to surgical complications in patients before ESS [4].

The lateral lamella of the cribriform plate (LLCP) and the posterior ethmoid roof (ER) are predominantly susceptible to CSF leaks due to their delicate bone composition [5-7]. The Keros classification is a widely utilized clinical tool for assessing the depth of the cribriform fossa, categorizing it based on the vertical separation between the ER and the cribriform plate (CP) into three distinct types. It implies that an elongated CP is more prone to damage [7,8]. Nonetheless, the Keros system has limitations in gauging the risk of injuries to the skull base due to its inherent curvature. Consequently, evaluating the Gera classification is advisable. According to the Gera methodology, the angle created by the lateral lamella (LL) and the horizontal plane originating from the CP is measured to determine the slope of the ER in relation to the CP, which is classified into three risk categories: Class I (>80°, low risk), Class II (45°-80°, moderate risk), and Class III (<45°, high risk) [7,9].

Penetrating the vertical basal lamella can easily compromise a low posterior ER, potentially leading to a CSF leak, injuries within the cranium, or damage to the optic nerve. Therefore, an assessment of the posterior ER's vertical elevation is crucial. Incorrect entry into the basal lamella can damage this region, even without an unusually low posterior ER [10]. The risk of skull base injury can be reduced by employing a low and medial approach to penetrate the posterior ethmoids via the basal lamella instead of utilizing a higher vertical entry point [11].

Ocular injuries, such as damage to the orbit, can occur because of bleeding from the anterior ethmoidal artery (AEA). A ruptured AEA can result in orbital or intracranial hematomas if drawn into the orbit or cranium. A review of preoperative CT imaging is also critical to evaluate the AEA location, especially floating or non-floating, for preventing these complications [12].

This retrospective study investigated the relationships among the Keros classification, the Gera classification, the vertical height of the posterior ER, and the AEA types in Japanese patients. Recognizing the anatomical nuances present, we hypothesized that the distinctive anatomical variations found among the Japanese significantly influence both the risk and the outcomes of ESS. In alignment with previous findings, such as those reported by Makihara et al., which indicated that the ethmoid sinus may be narrower in East Asians compared to Caucasians, thus potentially increasing the difficulty of ESS, particularly within the ethmoid sinus and frontal recess areas, our study seeks to deepen the surgical understanding and enhance risk assessment [13]. By examining these anatomical relationships, our goal is to contribute to the optimization of surgical techniques, achieve safer surgical practices, and provide a comprehensive view of the anatomical factors that may elevate surgical risks in Japanese patients.

## Materials and methods

A retrospective review was conducted of adult individuals aged ≥18 who had obtained a CT scan of their paranasal sinuses before undergoing ESS in the Department of Otolaryngology-Head and Neck Surgery at the Kagawa Rosai Hospital from March to November 2021. We excluded individuals who had previously undergone ESS or had a history of sinonasal tumors, skeletal dysplasia, or blowout fractures. The study adhered to the ethical guidelines in the Declaration of Helsinki and received approval from the ethical committee of the Kagawa Rosai Hospital (R2-28). The need to obtain informed patient consent for data use was waived owing to the retrospective nature of the study.

Before surgery, each patient underwent a multiplanar CT (Aquilion CT scanner, Toshiba Medical Systems, Tokyo, Japan) of the nasal cavity and adjacent paranasal sinuses with adjacent axial cross-sections of 0.5 mm thicknesses, similar to a previous report [12]. The acquired CT data were subsequently processed to generate sagittal and coronal image reconstructions on a specialized computer workstation.

**Figure 1:**
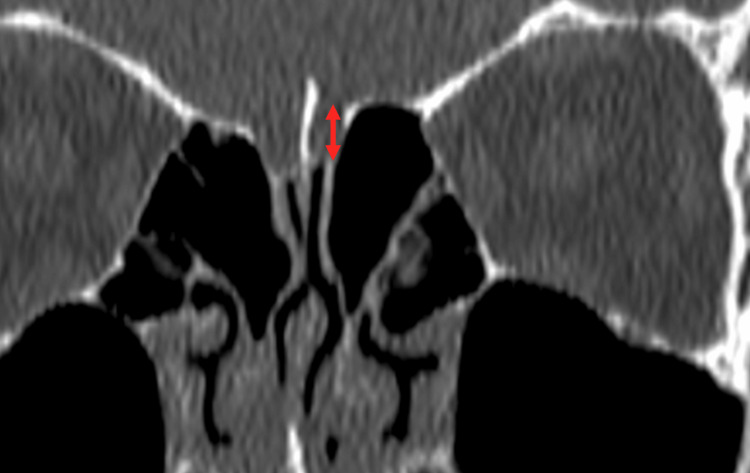
Depth of the cribriform plate measured as the largest vertical height of the olfactory fossa in the coronal plane

**Figure 2:**
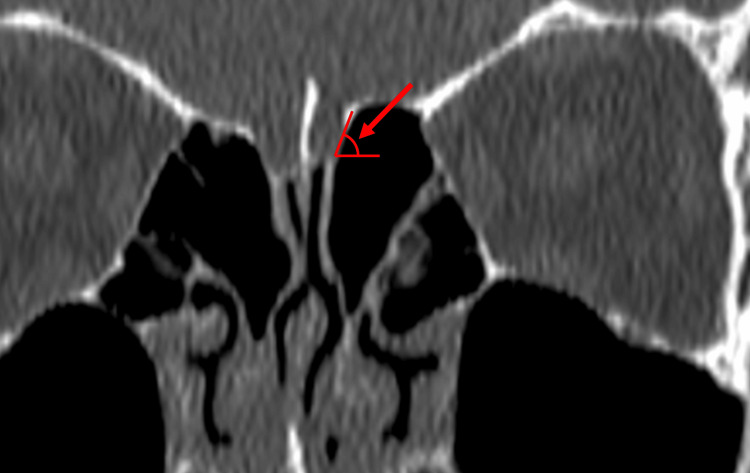
Measurement of the angle formed by the lateral lamella of the cribriform plate and the continuation of the horizontal plane passing through the cribriform plate

**Figure 3:**
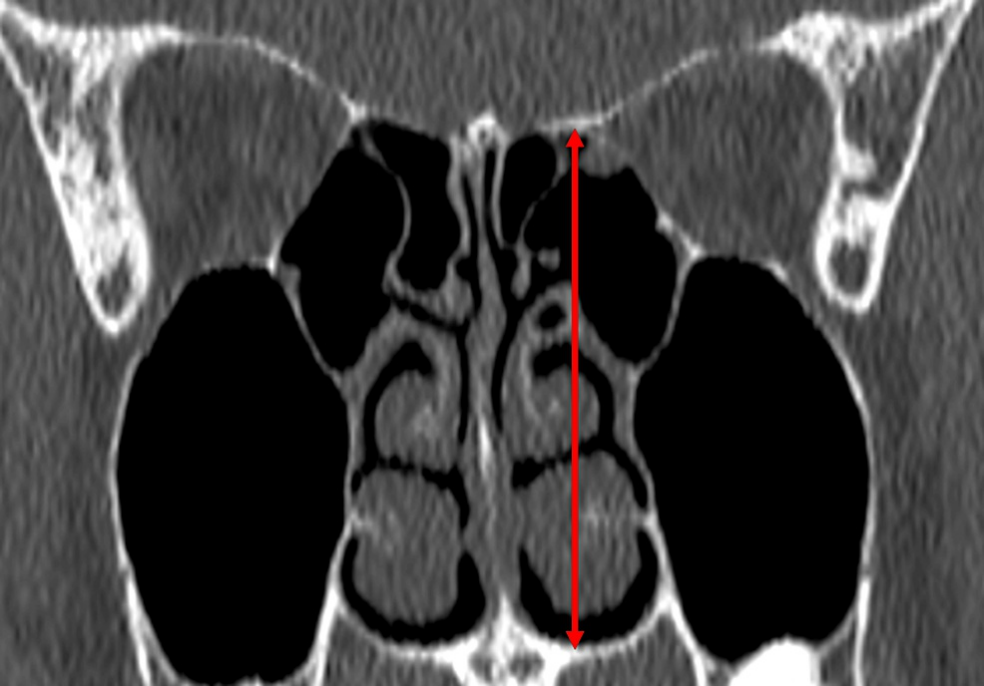
Measurement of the vertical height of the lateral ethmoid roof from the hard palate at the coronal plane of the posterior ethmoidal artery

Measurements were carried out in three distinct sets. The first set of measurements focused on determining the CP depth, specifically the maximal vertical elevation of the olfactory fossa in the coronal plane on both sides (Figure 1). According to the existing Keros classification system, Type I is characterized by a depth ranging from 1 to 3 mm, Type II extends from 4 to 7 mm, and Type III exceeds 7 mm in depth [8]. Discrepancies in depth between the right and left CPs were documented, specifically noting instances where the Keros classifications varied between the two sides. The second set of measurements focused on the LLCP angle (LLCPA), taken at the same coronal plane level as the CP measurements. This angle is constituted by the extension of the horizontal plane traversing the CP and the LLCP and was measured on both sides (Figure 2). Risk classifications according to the Gera system were assigned as follows: Class I signified an angle greater than 80° (low risk), Class II represented angles between 45° and 80° (medium risk), and Class III denoted angles less than 45° (high risk for skull base injury) [6]. In the third series of measurements, the maximal vertical elevation of the lateral ethmoid roof height posteriorly (LERHP) was gauged in the coronal plane corresponding to the posterior ethmoidal artery (PEA) on both sides (Figure 3).

The AEA has two courses: non-floating and floating. We conducted a subgroup analysis based on gender to explore these potential differences.

Two otolaryngologists (SM and TN) were responsible for performing the CT image measurements. To ensure consistency and standardization in our methodology, all cases were discussed in a preoperative conference, during which the measurements of each parameter for the surgery were performed and agreed upon.

Statistical analysis

The results are presented as mean with standard deviation. For assessing discrepancies in categorical variables, Fisher's exact test was deployed. The Mann-Whitney U test was utilized to scrutinize variations in continuous variables across different groups. Pearson's correlation analysis was employed to ascertain the relationship between the measured variables. P<0.05 was designated as the threshold for statistical significance. All statistical evaluations were conducted using the EZR (Easy R) software (Jichi Medical University Saitama Medical Center, Saitama, Japan) [14].

## Results

Between March and November 2021, 92 adult individuals underwent paranasal sinus surgical procedures at Kagawa Rosai Hospital's Department of Otolaryngology-Head and Neck Surgery. We excluded 32 patients with previous ESS, sinonasal tumors, or blowout fractures. Morphological evaluations were performed on the CT images of the remaining 60 Japanese patients (120 sides). The study group comprised 39 males (65.0%) and 21 females (35.0%). Participants' ages ranged from 18 to 81 (54.9±16.2) years.

Table 1 presents the classifications based on Keros and Gera, AEA types, CP depth, LLCPA, and the LERHP's height according to sex and side.

**Table 1:** Distribution of the Keros classifications, Gera classifications, type of AEA, depth of the CP, LLCPA, and height of the LERHP by side and sex P-values are for the association with side AEA, anterior ethmoidal artery; CP, cribriform plate; LLCPA, lateral lamella CP angle; LERHP, lateral ethmoid roof from the hard palate; ns, not significant; SD, standard deviation

	Right side	Left side	P-value	Total
Keros classification, n (%)				
Type I	21 (17.5)	15 (12.5)	ns	36 (30)
Type II	34 (28.3)	40 (33.3)		74 (61.7)
Type III	5 (4.2)	5 (4.2)		10 (8.3)
Total	60 (50)	60 (50)		120 (100)
Gera classification, n (%)				
Class I	13 (10.8)	10 (8.3)	ns	23 (19.2)
Class II	44 (36.7)	49 (40.8)		93 (77.5)
Class III	3 (2.5)	1 (0.8)		4 (3.3)
Total	60	60		120 (100)
AEA type, n (%)				
Floating AEA	35 (29.2)	42 (35)	ns	77 (64.2)
Non-floating AEA	25 (20.8)	18 (15)		43 (35.8)
Total	60 (50)	60 (50)		120 (100)
Depth of the CP, mean (SD), mm	4.45 (1.41)	4.91 (1.47)	ns	
LLCPA, mean (SD), degree	67.87 (12.80)	69.56 (12.00)	ns	
Height of the LERHP, mean (SD), mm	49.20 (3.29)	49.55 (3.05)	ns	
	Male	Female	P-value	Total
Keros classification, n (%)				
Type I	22 (18.3)	14 (11.7)	ns	36 (30)
Type II	49 (40.8)	25 (20.8)		74 (61.7)
Type III	7 (5.8)	3 (2.5)		10 (8.3)
Total	78 (65)	42 (35)		120 (100)
Gera classification, n (%)				
Class I	16 (13.3)	7 (5.8)	ns	23 (19.2)
Class II	59 (49.2)	34 (28.3)		93 (77.5)
Class III	3 (2.5)	1 (0.8)		4 (3.3)
Total	78 (65)	42 (35)		120 (100)
AEA type, n (%)				
Floating AEA	48 (40)	29 (24.2)	ns	77 (64.2)
Non-floating AEA	30 (25)	13 (10.8)		43 (35.8)
Total	78 (65)	42 (35)		120 (100)
Depth of the CP, mean (SD), mm	4.75 (1.52)	4.55 (1.33)	ns	
LLCPA, mean (SD), degree	69.15 (12.50)	67.91 (12.27)	ns	
Height of the LERHP, mean (SD), mm	50.27 (2.77)	47.71 (3.19)	<0.001	

The average CP depth was measured as 4.68±1.45 mm, ranging from 1.8 to 7.6 mm. Based on the Keros classification, 36 sides (30.0%) were of Type I, 74 (61.7%) of Type II, and 10 (8.3%) of Type III. Notably, Type II was the most frequently observed category, succeeded by Types I and III. The Keros classification was not significantly different between the two sexes (p=0.82) or the two sides (p=0.48). CP depth asymmetry (the Keros classification on the two sides) was evident in 33.3% of patients. In 65% of these instances, the right side showed a lesser depth than the left.

The average LLCPA measured 68.72°±12.38°, ranging from 32.7° to 90.0°. By the Gera classification, 23 (19.2%) sides were of Class I, 93 (77.5%) of Class II, and four (3.3%) of Class III. Reflecting this distribution, Gera Class II was the most common, followed by Classes I and III. No significant variations in the angle classification were detected based on sex (p=0.78) or side (p=0.44).

LERHP's height at the PEA's coronal plane averaged 49.38±3.16 mm, ranging from 41.3 to 56.7 mm. A significant statistical association was found between LERHP's height and sex (p<0.001). LERHP's height in males averaged 50.3±2.7 mm and in females averaged 47.7±3.2 mm (Table 1). No significant variations were seen in LERHP's height between sides (p=0.50).

A positive association was evident between CP depth and LLCPA (r=0.63; p<0.01, as shown in Table 2). A slight positive link was also found between CP depth and LERHP's height (r=0.19; p<0.05). Table 3 delineates the classifications according to Keros and Gera for each side. The pair of Keros Type II with Gera Class II was most prevalent at 50%. Some combinations were not detected, such as Keros Type II with Gera Class III and Keros Type III with Gera Class III. A significant link between the Keros and Gera classifications was noted (p<0.05, as per Table 3).

**Table 2:** The correlations between CP depth, LLCPA, and the height of the LERHP (assessed by Pearson's coefficient, r) *Statistically significant at p≤0.05 (Pearson's correlation test) **Statistically significant at p≤0.01 (Pearson's correlation test) CP, cribriform plate; LLCPA, lateral lamella CP angle; LERHP, lateral ethmoid roof from the hard palate

	Depth of the CP	LLCPA	Height of the LERHP
Depth of the CP	1	0.63^**^	0.19^*^
LLCPA	-	1	0.15
Height of the LERHP	-	-	1

**Table 3:** Co-distribution of the Keros and Gera classifications Values are reported as n (%)

Keros classification	Gera classification				P-value
	Class I	Class II	Class III	Total	
Type I	1 (0.8)	31 (25.8)	4 (3.3)	36 (30.0)	<0.001
Type II	14 (11.7)	60 (50)	0 (0.0)	74 (61.7)	
Type III	8 (6.7)	2 (1.7)	0 (0.0)	10 (8.3)	
Total	23 (19.2)	93 (77.5)	4 (3.3)	120 (100.0)	

Sagittal CT scans showed 43 non-floating AEAs (35.8%) and 77 floating AEAs (64.2%) in the 120 examined sides. The AEA types did not differ significantly between sexes or sides (p=0.41 and p=0.18, respectively). As the Keros classification increased (indicating an increase in CP depth), floating AEA prevalence increased as well (p<0.001, as shown in Table 4). Conversely, an increase in the Gera classification (indicative of a decrease in LLCPA) increased the prevalence of non-floating AEAs (p=0.002, shown in Table 4).

**Table 4:** Association between the type of AEA and the Keros and Gera classifications Values are reported as n (%) AEA: anterior ethmoidal artery

Keros classification	AEA type			P-value
	Floating AEA	Non-floating AEA	Total	
	Side (%)	Side (%)	Side (%)	
Type I	12 (10)	24 (20)	36 (30)	<0.001
Type II	56 (46.7)	18 (15)	74 (61.7)	
Type III	9 (7.5)	1 (0.8)	10 (8.3)	
Total	77 (64.2)	43 (35.8)	120 (100)	

Gera classification	AEA type			P-value
	Floating AEA	Non-floating AEA	Total	
	Side (%)	Side (%)	Side (%)	
Class I (>80)	20 (16.7)	3 (2.5)	23 (19.2)	0.002
Class II (45-80)	57 (47.5)	36 (30)	93 (77.5)	
Class III (<45)	0 (0)	4 (3.3)	4 (3.3)	
Total	77 (64.2)	43 (35.8)	120 (100)	

## Discussion

This is an initial research to elucidate the correlation between the height of the LLCP and the posterior ER. Globally, the Keros classification serves as the standard for assessing LLCP elevation. In our Japanese patient cohort, Type II emerged as the most frequently observed category, succeeded by Types I and III. These findings align with previous research conducted in Saudi Arabia, India, Brazil, Korea, Turkey, the United States, and Italy, as indicated in Table 5 [6,8,15-20]. Conversely, research on Egyptian and Filipino demographics has shown Keros Type I to predominate, followed by Types II and III [21,22]. Such variations in the distribution of Keros types may be attributed to several factors, including ethnic differences, genetic predisposition, environmental influences, prior chronic infections, and sample size. Patients classified under Keros Type III are notably more susceptible to iatrogenic injuries to the skull base due to the increased depth of the cribriform cleft [23,24].

**Table 5:** Distribution of the Keros and Gera classifications among different studies

Author	Country	N	Keros classification			Asymmetry (%)
			Type I (%)	Type II (%)	Type III (%)	
Present study	Japan	60	30	61.7	8.3	33.3
Madani et al. [15]	Saudi Arabia	511	12.5	53.2	11.7	22.5
Nair [16]	India	180	17.2	77.2	5.6	11.7
Pawar et al. [17]	India	200	18.5	74.5	7.0	11.5
Souza et al. [18]	Brazil	200	26.2	73.3	0.5	12.0
Jang et al. [19]	Korea	205	30.5	69.5	0	-
Güler et al. [20]	Turkey	300	26	66	8	-
Ramakrishnan et al. [8]	United States	200	42	50	8	-
Gera et al. [6]	Italy	190	20	64.7	15.3	-
Shama and Montaser [21]	Egypt	100	56.5	40.5	3.0	-
Paber et al. [22]	Philippine	109	81.8	17.7	0.5	-

Author	Country	N	Gera classification		
			Class I (%)	Class II (%)	Class III (%)
Present study	Japan	60	19.2	77.5	3.3
Mahdian et al. [23]	Iran	372	29.6	61.4	9.0
Özeren Keşkek and Aytuğar [7]	Turkey	385	34.7	63	2.3
Abdullah et al. [9]	Malaysia	150	23.7	72.3	4.0
Fadda et al. [24]	Italy	220	17.7	77.5	4.8
Gera et al. [6]	Italy	190	32.6	62.7	4.7

In our current investigation, a depth disparity in the CP was observed in approximately one-third of the patient population (33.3%). In most instances, the CP depth was shallower on the right side than on the left. This observation concurs with findings from earlier studies [6,25]. Dessi et al. [25] have posited that the more frequent occurrence of CSF leaks on the right side, as indicated in the literature, might be linked to the generally lower elevation of the right ER relative to the left in numerous cases [26,27].

The present study showed that Gera Class II was the most common, followed by Classes I and III in Japanese patients, consistent with other studies in the Iranian, Turkish, Malaysian, and Italian populations (Table 5) [6,7,9,23,24]. The reason for the consistency in the proportion of Gera classes may be that the classification itself is new, and there are few reports from various countries.

Our current research identified a positive association between the depth of the CP and the angle at the LLCPA. This finding is corroborated by earlier studies that have also revealed a positive association between CP depth and LLCPA. This is in line with the work conducted by Elwany et al. [28], who posited that the olfactory fossa depth is contingent on the LL field's length and angular disposition [6,7,9]. According to the Keros classification framework, a shallower CP depth indicates a reduced risk, whereas a diminished LLCPA, as assessed by the Gera classification, signifies an elevated risk. This means that the surgeon must be careful not to penetrate superomedially instead of medially if the depth of the CP is less and the LLCPA is low. The Keros and Gera classifications have been suggested for complementary classifications in preoperative risk assessments of CSF leaks.

The posterior ER is also a high-risk site for CSF leak with LLCP [5-7]. LLCP and anterior ER are commonly assessed using the Keros and Gera classifications. However, a standardized classification for the posterior ER is currently lacking. In a study by Ramakrishnan et al., a proportion was derived by comparing the maximum vertical height of the maxillary sinus in the largest coronal plane with the corresponding vertical height of the ethmoid in the same plane. This ratio, termed maxillary to ethmoid height ratio (MER), was categorized into three distinct groups: Type I (1:1), Type II (2:1), and Type III (greater than 2:1) [8]. It was posited that a skull base associated with the highest ratio (Type III) would be situated lower and, consequently, more susceptible to injury, categorizing it within the high-risk bracket. Baban et al. reported that MER was measured at the point of the posterior ethmoid [5]. The vertical dimension of the maxillary sinus at its peak is occasionally gauged at the anterior ethmoid sinus. The PEA supplies the nasal septum, posterior ethmoid cells, and the dura that overlies the planum sphenoidale. This artery is proximal to the junction of the roof of the sphenoid sinus within the posterior ethmoid sinus [29].

The PEA is the first transverse canal in coronal sections viewed posteriorly to anteriorly from the planum sphenoidale [29]. Therefore, we recorded the vertical height of the LERHP in the coronal plane of the PEA as the basis for the posterior ethmoid sinus. The present study found a weak positive correlation between CP depth and LERHP height. In this study, some patients had a low height of the LERHP, which is assessed as a high-risk factor for CSF leak at the posterior ER, even though they only had Keros Types I and II. Therefore, examining the LERHP height in the PEA's coronal plane with the Keros classification provides additional information to reduce the risk of a CSF leak. Given the weak positive correlation observed between CP depth and LERHP height, we treated these variables as independent factors in our analysis. This distinction is critical because each factor contributes uniquely to the overall risk assessment. Therefore, in the context of preoperative planning, the Keros classification and LERHP height should be interpreted as complementary elements. This combined approach enriches the evaluation process, facilitating a more comprehensive assessment of the risk for CSF leaks during ESS. The vertical height of the LERHP in females was significantly lower than that in males; however, there was no significant difference in the distribution of the Keros and Gera classifications between female and male patients. Low posterior ethmoid sinus height can lead to CSF leak while penetrating the basal lamella, and surgeons should pay careful attention to this aspect, especially in females. The observed difference in the vertical height of the LERHP between females and males may be attributable to inherent anatomical variations linked to sex-specific physical characteristics. There are general differences in the craniofacial morphology between females and males. These differences could potentially influence the anatomy of the paranasal sinuses and the skull base. Females generally have smaller cranial sizes, which might result in a lower height of the LERHP, thereby increasing the risk of complications such as CSF leaks during ESS.

The frequency of the occurrence of a floating AEA increased in conjunction with the depth of the CP, a finding that aligns with a recently disseminated review (p<0.001) [30]. This review indicated that the AEA's position along the skull base is influenced by the LLCP's height and the manifestation of supraorbital ethmoid cells. With an average thickness of merely 0.2 mm, the LLCP can be further attenuated to a mere 0.05 mm at the specific juncture where the AEA traverses the LLCP to access the anterior cranial fossa. This represents the most fragile point on the skull base and is frequently the site of CSF leaks [30]. Since there are many situations in which the AEA is floating due to a long LLCP, surgeons must be careful to avoid orbital hemorrhage due to AEA damage and avoid a CSF leak at the point on the LLCP where the AEA enters. However, with the increase in the Gera classification (decrease in LLCPA), the prevalence of non-floating AEAs also increased (p=0.002). This study is the first to report the relationship between the Gera classification and the occurrence of floating AEAs. The AEAs may be located adjacent to the skull base, particularly when the ER is low [30]. Conversely, in situations where the CP is elongated, which is associated with a higher ER, it can be hypothesized that the AEA tends to be floating. This anatomical arrangement may be a result of the increased distance between the AEA and the skull base in cases of a higher ER. With the increase in the Gera classification (decrease in LLCPA), the risk of a CSF leak in the area where the AEA enters the CP should be noted rather than an orbital hemorrhage from AEA bleeding because the AEA is often not floating.

This study had certain limitations because it was performed at a single institution. First, there was no risk classification for the LERHP's height in the PEA's coronal plane. Similar to the Keros or Gera classification, a new standardized primary classification of the height of the posterior LERHP could help reduce CSF leaks by allowing preoperative risk management. Second, the study's sample size was small compared to previous reports on ER classifications. Due to the limited sample size and single-institution nature of the study, the findings should be interpreted with caution and not be generalized to the general population.

Fortunately, we have not encountered any cases of iatrogenic anterior skull base injuries during surgery in our hospital. As we continue to accumulate more cases through a multicenter study with a large sample size, we anticipate that if we do encounter such injuries, comparing them to non-injury cases could be instrumental in developing and validating a new standardized primary classification for the height of the posterior LERHP. This approach would allow us to identify specific indicators within this classification system. Furthermore, we believe that such comparative analysis would be crucial in proving the validity of this classification as a risk assessment tool, thereby enhancing the precision and safety of ESS. We have drawn correlations between CP depth and the risk of CSF leaks or AEA-related complications, but it is important to emphasize that these findings suggest an association, not a definitive cause-and-effect relationship. The methods for assessing these risk factors are not yet fully developed. Therefore, further research is essential, including the aforementioned studies that compare cases with and without anterior skull base injuries. At this stage, it is imperative to combine existing methods of risk assessment and conduct a thorough preoperative evaluation for each individual case. In line with this, within surgical training programs, it is essential for mentors to diligently engage with trainees in preoperative CT interpretation. This hands-on approach not only reinforces the understanding of these complex anatomical variations but also emphasizes the critical importance of detailed preoperative planning.

## Conclusions

The low height of the posterior ER, where CSF leaks occur while penetrating the basal lamella, is often evident in females, and the height positively correlates with the Keros classification in Japanese patients. The Keros and Gera classifications, AEA type, and posterior ER height cannot help complete risk assessment in isolation. Nevertheless, when used together, they may correlate and increase the likelihood of preventing significant complications, such as CSF leaks and orbital hemorrhage.
